# Strain-Controlled Galvanic Synthesis of Platinum Icosahedral Nanoframes and Their Enhanced Catalytic Activity toward Oxygen Reduction

**DOI:** 10.1021/acs.nanolett.4c02764

**Published:** 2024-10-18

**Authors:** Siyu Zhou, Minghao Xie, Yong Ding, Zhiqi Wang, Quynh Nguyen, Kei Kwan Li, Younan Xia

**Affiliations:** † School of Chemical and Biomolecular Engineering, Georgia Institute of Technology, Atlanta, Georgia 30332, United States; ‡ School of Chemistry and Biochemistry, Georgia Institute of Technology, Atlanta, Georgia 30332, United States; # School of Materials Science and Engineering, Georgia Institute of Technology, Atlanta, Georgia 30332, United States; § The Wallace H. Coulter Department of Biomedical Engineering, Georgia Institute of Technology and Emory University, Atlanta, Georgia 30332, United States

**Keywords:** strain engineering, galvanic replacement, site selectivity, icosahedral nanoframe, oxygen reduction

## Abstract

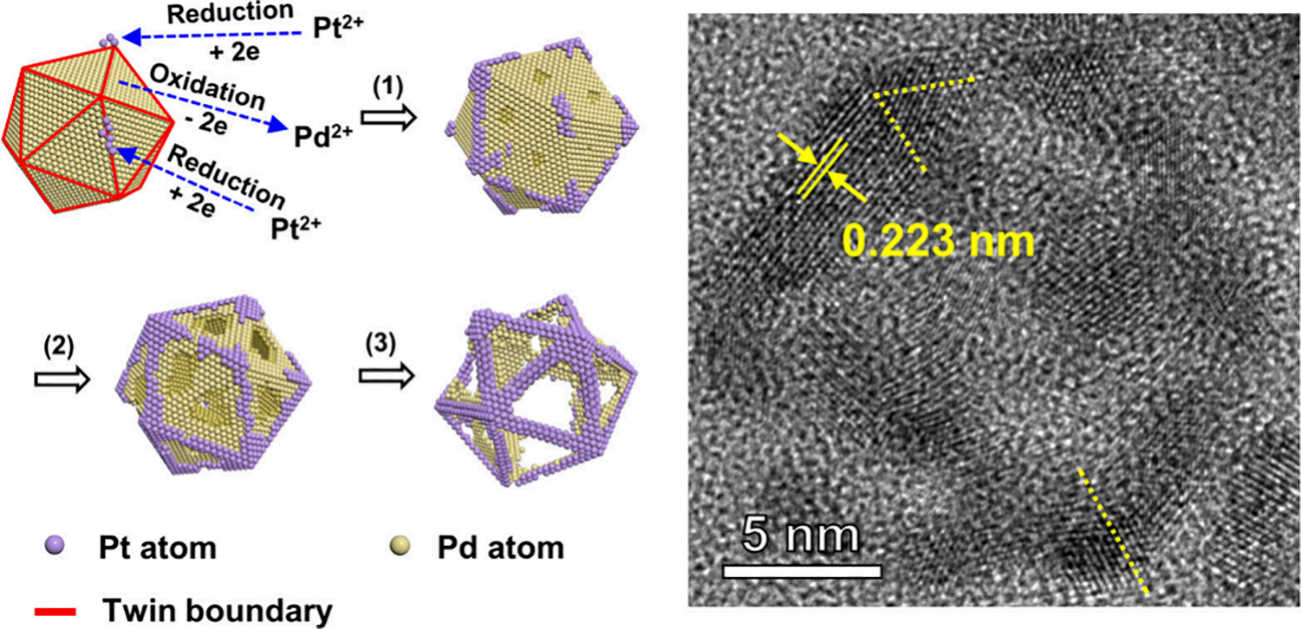

The unique strain distribution on the surface of a Pd icosahedral nanocrystal is leveraged to control the sites for oxidation and reduction involved in the galvanic replacement reaction. Specifically, Pd is oxidized and dissolved from the center of each {111} facet due to its tensile strain, while the Pt(II) precursor adsorbs onto the vertices and edges featuring a compressive strain, followed by surface reduction and conformal deposition of the Pt atoms. Once the galvanic reaction is initiated, the {111} facets become more vulnerable to oxidation and dissolution, as the vertices and edges are protected by the deposited Pt atoms. The site-selected galvanic reaction naturally results in the formation of Pt icosahedral nanoframes covered by compressively strained {111} facets, which show enhanced catalytic activity and durability toward oxygen reduction relative to commercial Pt/C.

Galvanic replacement offers a facile and versatile route to the synthesis of nanostructures with well-controlled compositions and morphologies for a variety of applications related to catalysis, electrocatalysis, imaging, and sensing.^1−17^ While most of the studies reported in the literature involved the use of Ag nanocrystals, our group demonstrated that Pd nanocubes also underwent galvanic replacement with PtCl_6_^2–^ in the presence of Br^–^ for the generation of Pd–Pt concave nanocubes.^18^ The Pd nanocubes were slightly truncated at corners and thus enclosed by a mixture of {100} and {111} facets. Due to their coverage by the chemisorbed Br^–^ ions, the {100} facets were preferentially oxidized and etched away during galvanic replacement, while the Pt atoms derived from PtCl_6_^2–^ ions were selectively deposited on the vertices terminated in {111} facets. The as-obtained Pd–Pt concave nanocubes showed enhanced activity toward oxygen reduction relative to commercial Pt/C. Due to the elimination of self-nucleation, galvanic replacement offers immediate advantages over seed-mediated growth, another method commonly used for the synthesis of nanocrystals with well-controlled compositions and morphologies.^19,20^ The simplicity typical of a galvanic synthesis also makes this approach viable for reproducible and scalable production.

The site selectivity in the aforementioned example is instrumental in the fabrication of nanostructures with well-defined and controllable morphologies. To date, only a few strategies have been explored to achieve the site selectivity of a galvanic replacement, limiting the types of nanostructures being produced. In the above example, the facet selectivity of a capping agent is responsible for the site-selective galvanic replacement.^21^ Specifically, Br^–^ ions bind most strongly to the Pd{100} facets for the initiation of selective oxidation and dissolution of Pd from these facets while sparing the Pd{111} facets for Pt deposition. In general, this approach only works for nanocrystals whose surface is selectively capped by a ligand, and it would be difficult to perform site-selective galvanic replacement on nanocrystals solely enclosed by one type of facet or nanocrystals free of a strongly binding ligand.^22^

Herein, we demonstrate that the surface strain on a Pd icosahedral nanocrystal can be utilized to control the sites for oxidation and reduction involved in a galvanic replacement reaction with PtCl_4_^2–^. As a multiply twinned nanocrystal, each icosahedron can be considered as an assembly of 20 identical tetrahedral units.^23,24^ During the formation of an icosahedron, twin boundaries are introduced between neighboring components, and the lattice of each tetrahedral unit must be stretched in order to close the gap left behind by the packing of the tetrahedral units (Figure S1). As such, there is a tensile strain in the center of each facet, while the vertices and edges experience a compressive strain (Figure S2). The presence of the tensile strain and compressive strain has been confirmed by analyses involving atomic-resolution high-angle annular dark-field scanning transmission electron microscopy (HAADF-STEM).^23,25,26^ According to theoretical calculations, the tensile strain at the center of each {111} facet on a Pd icosahedron gradually decreases along the radial direction and transitions to a compressive strain at the vertices and edges.^23^ During the galvanic replacement, the oxidation and dissolution of Pd will be preferentially initiated from the center of each {111} facet due to the tensile strain and thus the higher chemical potential for the Pd atoms. Meanwhile, the Pt atoms derived from the reduction of PtCl_4_^2–^ are conformally deposited at the vertices and edges. Once the vertices and edges are fully protected by the deposited Pt atoms, the {111} facets become more susceptible to further oxidation and dissolution. When all of the Pd atoms in the interior have been etched away, a Pt icosahedral nanoframe will be left behind. Since the Pt nanoframe features the compressively strained {111} facets on the vertices and edges of a Pd icosahedron, it exhibits enhanced catalytic activity toward oxygen reduction reaction (ORR) relative to commercial Pt/C.^27^

Figure 1a shows a transmission electron microscopy (TEM) image of Pd icosahedra with an average size of 20 nm. The “size” refers to the dimension “*l*” defined in Figure S3, which corresponds to the distance between two opposite side faces. When viewed under TEM, icosahedra may exhibit different profiles, depending on their orientations relative to the electron beam. The magnified TEM image in the inset of Figure 1a shows an icosahedron oriented with its 2-fold axis oriented parallel to the electron beam.

**Figure 1:**
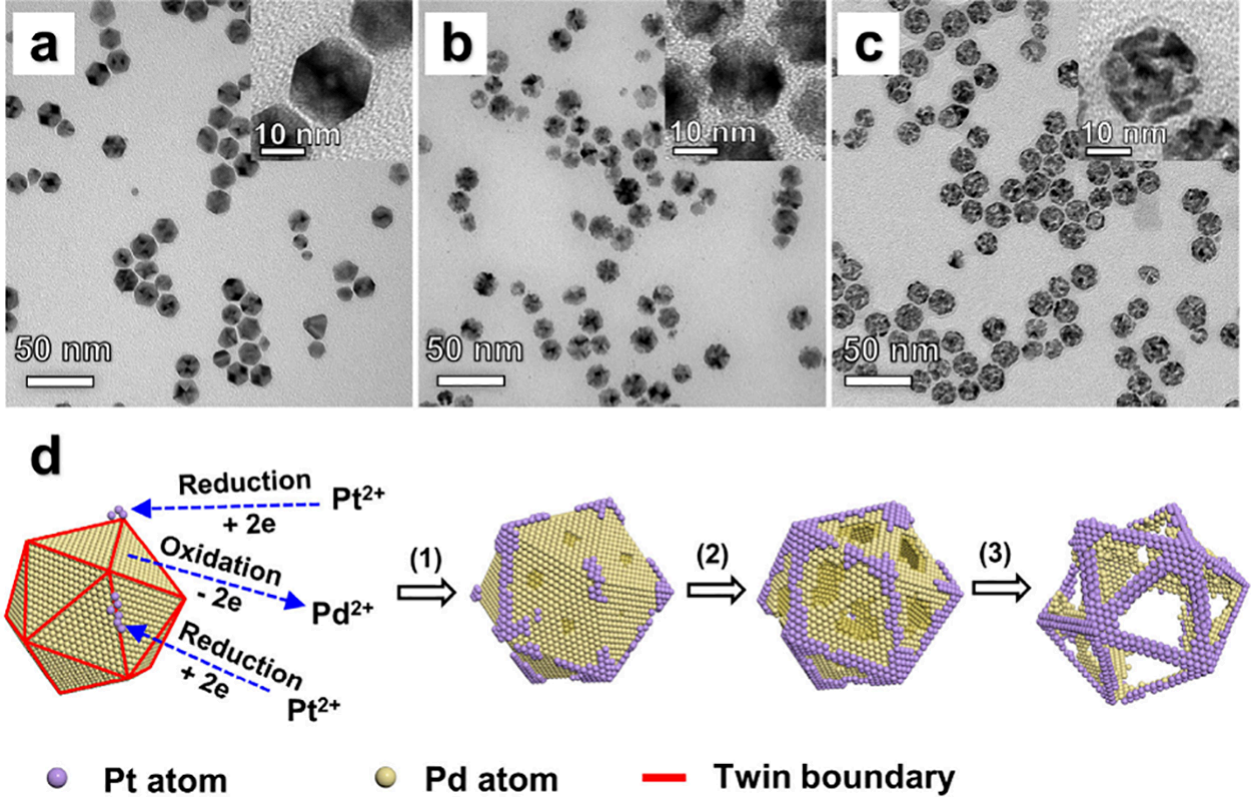
Morphological evolution of Pd icosahedra during their galvanic replacement reaction with K_2_PtCl_4_. (a–c) TEM images of the samples obtained using the standard protocol except for the difference in reaction time: (a) 0, (b) 1, and (c) 15 h. (d) Schematic showing the galvanic replacement between a Pd icosahedron and K_2_PtCl_4_ and the detailed morphological evolution. The red lines indicate the twin boundaries located at the edges of the icosahedron, which are gradually covered by the newly formed Pt atoms during the reaction.

In a typical synthesis, aqueous K_2_PtCl_4_ was mixed with the Pd icosahedra in an aqueous medium to initiate the galvanic replacement reaction. With KBr at a high concentration, added PtCl_4_^2–^ should undergo ligand exchange to form PtBr_4_^2–^ due to a significantly higher stability of the latter complex. Because the reduction potential of PtBr_4_^2–^/Pt (0.70 V versus RHE) is greater than that of PdBr_4_^2–^/Pd (0.49 V versus RHE), Pd would be spontaneously oxidized by Pt(II).^28^ As such, the galvanic reaction between Pd and Pt(II) is supposed to take place according to the following equation:^18^
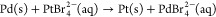


The galvanic replacement reaction can be broken down into two half reactions: oxidization and corrosion of Pd and reduction of PtBr_4_^2–^, followed by deposition of Pt on the Pd icosahedra. These two half reactions might occur at different sites on a nanocrystal due to the difference in strain and thus chemical potential or reactivity for the atoms at these sites.

We monitored the morphological evolution of the Pd icosahedral nanocrystal by analyzing the TEM images of the products sampled at different time points. After the reaction had proceeded for 1 h (Figure 1b), the {111} facets were excavated while the vertices and edges were largely unchanged, indicating that the dissolution of Pd atoms started from the facets, while both the vertices and edges were preserved. Our inductively coupled plasma mass spectrometry (ICP-MS) data confirmed the presence of Pt and show a Pt to Pd elemental ratio of 0.65 (Table S1). As the cavity in the Pd icosahedron continuously grew in size, only the vertices and edges remained, and a nanoframe-like structure of roughly the same size as the original Pd icosahedron was obtained when the reaction further proceeded to *t* = 15 h (Figure 1c). Our EDX analysis further confirmed that the as-obtained nanoframes were composed of both Pt and Pd, with a Pt to Pd elemental ratio of 1.04 according to the ICP-MS data. The Pd remaining in the nanoframes can be attributed to the fact that the molar ratio of the Pt in the precursor to the Pd contained in the icosahedra was controlled to be 1:2 to help generate Pt nanoframes’ thin ridges for oxygen reduction.^29,30^Figure 1d shows a schematic that details the morphological changes of a Pd icosahedron during the galvanic synthesis and the site selectivity for Pd dissolution and Pt deposition. The overall dimensions of the nanoframes could be controlled by varying the sizes of the Pd icosahedra. For example, nanoframes of about 10 nm in size were also synthesized by switching to 10 nm Pd icosahedra (Figure S4).

The presence of KBr is critical for initiating the galvanic replacement reaction and, thus, the formation of nanoframes. We also performed control experiments in the absence of KBr, while maintaining all other parameters. Under these conditions, etching of the Pd icosahedra was negligible, and the original solid structure was preserved (Figure S5). The exact morphology of the nanoframes can be varied by adjusting the amount of Pd icosahedra involved. We first reduced the particle concentration to half of the level used in the standard protocol, where the molar ratio of Pt in the precursor to Pd in the icosahedral template was set to 1:2. At the reduced Pd content, the ridge thickness of the resultant nanoframes decreased due to the lower content of residual Pd, but the frame-like structure was maintained (Figure S6a). Conversely, we doubled the concentration of Pd icosahedra to increase the content of the residual Pd. In this case, the products looked less frame-like, although they remained hollow (Figure S6b).

Many factors may contribute to the site selectivity observed for the galvanic replacement reaction, with the most prominent one being the involvement of a surface capping agent.^3^ In the aforementioned example of the galvanic replacement between slightly truncated Pd nanocubes and H_2_PtCl_6_, the resultant Pt atoms were selectively deposited on the {111} facets because the {100} facets were capped by Br^–^ ions, which had oxidative etching capability. Since Pd icosahedra are free of surface capping agents, the site selectivity should be ascribed to other factors, in this case, variance in the coordination numbers, the twin defects contained in the edges, or the nonuniform strain distribution on the surface. First, the edges have lower coordination number and contain twin defects, making them susceptible to oxidation and etching.^31−34^ In the present case, however, it was the center of the facets rather than the vertices or edges that was preferentially oxidized and dissolved. We argued that the surface strain can also play a critical role in altering the chemical potential of surface atoms and thus control the site selectivity for the galvanic reaction on Pd icosahedra. Specifically, the compressive strain on the vertices and edges of the icosahedron would lower the chemical potential of these sites, while the tensile strain on the facets would increase the chemical potential. As a result, the atoms situated at the center of each facet are more susceptible to etching and dissolution, sparing the vertices and edges for Pt deposition.

As expected, replacing the Pd icosahedra with nanocrystals in other shapes may change the preferential site for galvanic replacement due to their differences in strain distribution, resulting in the formation of different nanostructures. For example, cubes and octahedra would differ from their icosahedral counterparts because of their more or less uniform distribution in strain across the surface. In these two cases, one has to rely on other means to induce site selectivity for galvanic replacement. Specifically, the {100} facets on Pd cubes could be made more susceptible to etching through Br^–^ adsorption, forcing Pt deposition toward the {111} facets to generate a cage-like structure.^18^ Here we also experimentally investigated the case of octahedra by replacing Pd icosahedra with the same amount of Pd octahedra. As expected, Pd was preferentially etched away from the {100} facets, resulting in products with a cage-like structure (Figure S7).

We used high-resolution TEM (HRTEM), scanning TEM (STEM), and energy dispersive X-ray (EDX) spectroscopy to systematically characterize the Pt–Pd icosahedral nanoframes. As shown by Figure 2a, the Pt–Pd nanoframes still had an icosahedral shape, with an overall size similar to that of the Pd icosahedra. The STEM image in Figure 2b confirms the frame-like structure of each particle. Figure 2c shows an HRTEM image taken from an individual nanoframe, with a model in the inset to depict its orientation relative to the electron beam. Two regions of the nanoframe were selected for further characterization (Figure 2, parts d and e). The well-resolved twin defects (marked by yellow dotted lines) indicate that the nanoframe replicated the twin-lined vertices and edges of a Pd icosahedron, primarily due to the match in lattice constant between Pd and Pt and thus epitaxial growth of Pt during the galvanic replacement reaction.^19^ The presence of twin defects was confirmed by their corresponding FFT patterns (Figure S8). Similar to the Pd template, the Pt atoms deposited at/near the twin boundaries experience a compressive strain (Figure 2d).

**Figure 2:**
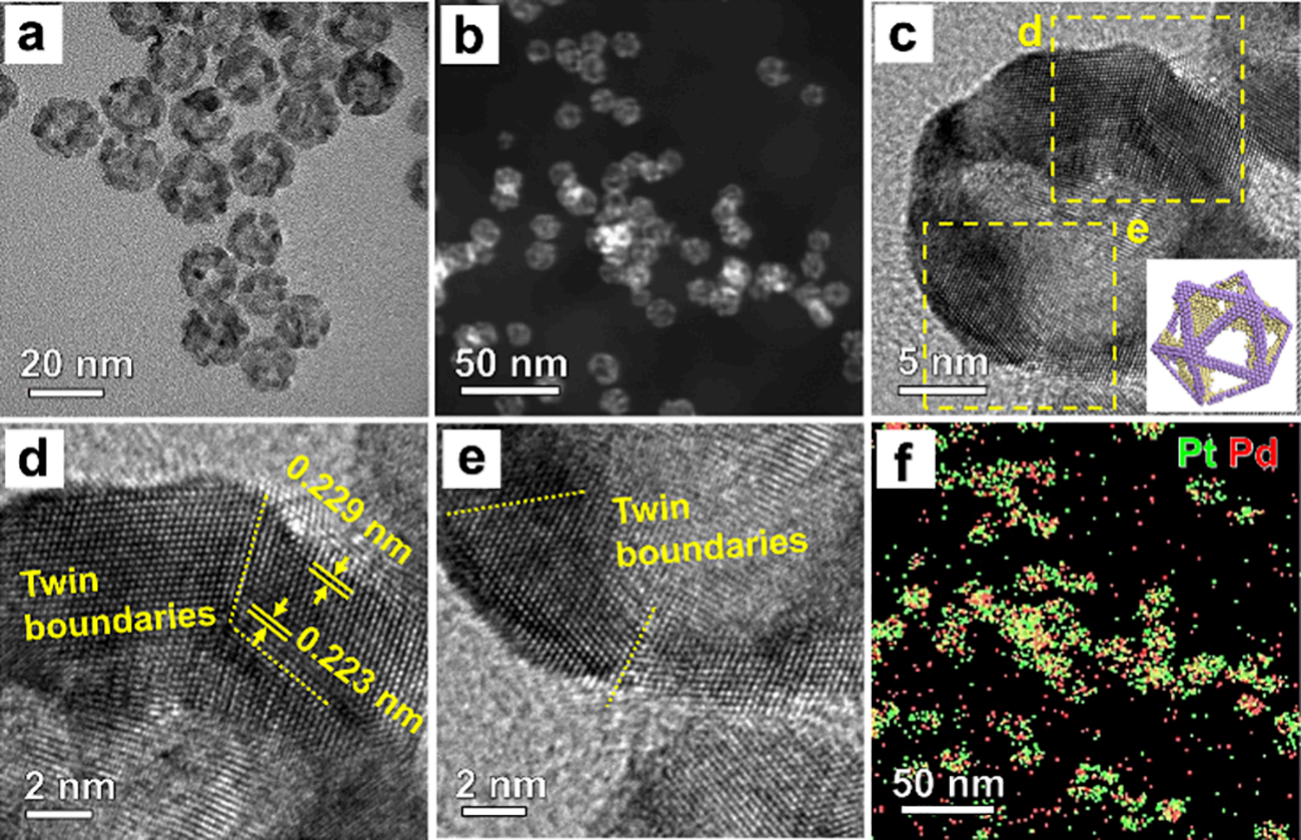
(a) TEM and (b) STEM images of the Pt–Pd icosahedral nanoframes. (c) HRTEM image taken from a single nanoframe. (d, e) HRTEM images taken from the areas marked by the boxes in (c). Note that the interplanar distance of {111} facets near twin boundaries was reduced relative to bulk Pd, while the value for planes in other regions was similar to the equilibrium state. (f) EDX mapping of Pd and Pt elements in the nanoframes from the area shown in (b).

We analyzed the elemental composition and distribution of the as-prepared icosahedral nanoframes by EDX mapping. Figure 2f shows the overlap in color between Pt (green) and Pd (red), confirming a bimetallic structure. Moreover, the spatial distribution of Pt and Pd differs on an icosahedral nanoframe. This can be attributed to the fact that the interdiffusion between Pd and Pt was not prominent at a relatively low reaction temperature of 90 °C. Significant interdiffusion and generation of a Pd–Pt alloy should not occur until the temperature reaches 400 °C.^35,36^ At the end, residual Pd could be removed by etching with an aqueous solution containing FeCl_3_ and KBr, generating Pt icosahedral nanoframes. When the residual Pd was mostly etched away, the particles still kept their frame-like structure and the original particle size while the ridges became thinner (Figure 3a). Moreover, the HRTEM image and the corresponding fast Fourier transform (FFT) pattern clearly show the twin defects and compressively strained {111} facets in the nanostructure obtained after etching (Figures 3b and S9). The retention of compressive strain in the nanoframes is likely due to the presence of twin defects.^37^ These defects cause the lattice to adjust to new boundary conditions while striving to maintain the overall structural integrity. As a result, atoms near the twin boundary may be forced closer than those in their positions in a bulk sample. Our previous theoretical calculations have demonstrated that Pt nanocrystals with a pent-twinned structure also experience a compressive strain.^27^ The STEM image confirms the hollow frame-like structure of the particles (Figure 3c). The ICP-MS results indicated that most of the residual Pd had been removed and the final products only contained 15 mol % of Pd, which is consistent with the result from EDX mapping (Figure 3d). We made catalysts based on the Pt icosahedral nanoframes shown in Figure 3 and tested their activity and durability toward the ORR.

**Figure 3:**
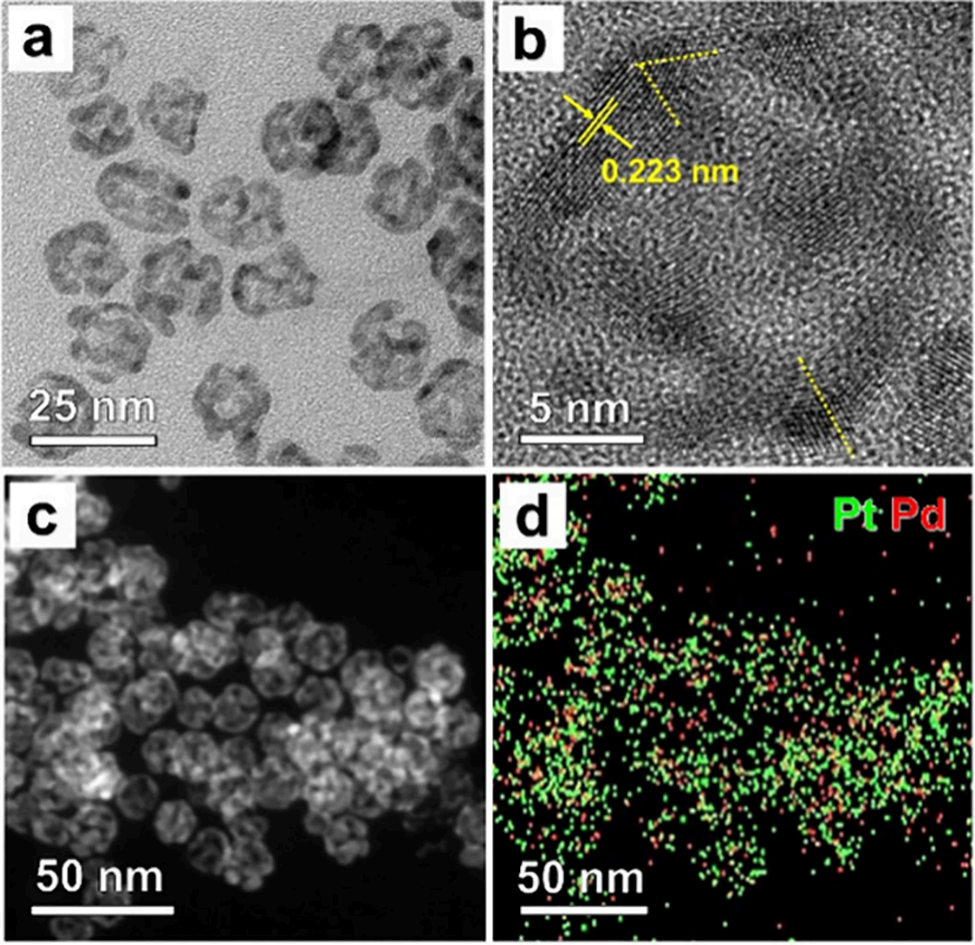
(a) TEM, (b) HRTEM, and (c) STEM images of the sample after removing more Pd from the as-obtained Pd–Pt nanoframes shown in Figure 2. (d) EDX mapping of Pd and Pt elements in the sample from the area shown in (c). The yellow dashed lines in (b) represent twin boundaries.

We benchmarked the electrocatalytic properties of the Pt icosahedral nanoframes toward the ORR against a commercial Pt/C catalyst (Figure 4). To determine their electrochemical active surface areas (ECSAs), we recorded cyclic voltammograms (CVs) of the catalysts in a 0.1 M HClO_4_ solution (Figure 4a). The ECSAs were derived by calculating the charges associated with hydrogen adsorption (H_upd_) and assuming 240 μC/cm^2^ (for nanoframes) or 210 μC/cm^2^ (for commercial Pt/C) for the adsorption of a monolayer of hydrogen on the Pt surface.^38,39^ We then determine the specific ECSA by normalizing the value to the mass of Pt in the sample. Notably, the specific ECSA of the Pt icosahedral nanoframes was as high as 115.2 m^2^ g^–1^_Pt_, which was nearly twice that (69.4 m^2^ g^–1^_Pt_) of commercial Pt/C (Table S2). Although the Pt nanoframes had a relatively overall size as large as 20 nm, they had an ultrathin thickness of 2 nm for their ridges (Figure 3). As such, a larger proportion of the Pt atoms in the nanoframes was exposed on the surface, including both the outer and inner surfaces, to participate in the electrocatalytic reaction, leading to a marked increase in ECSA relative to that of Pt/C. Moreover, the nanoframes were well dispersed on the carbon support without aggregation, which could have also contributed to the high specific ECSA of the Pt nanoframes (Figure S10).

**Figure 4:**
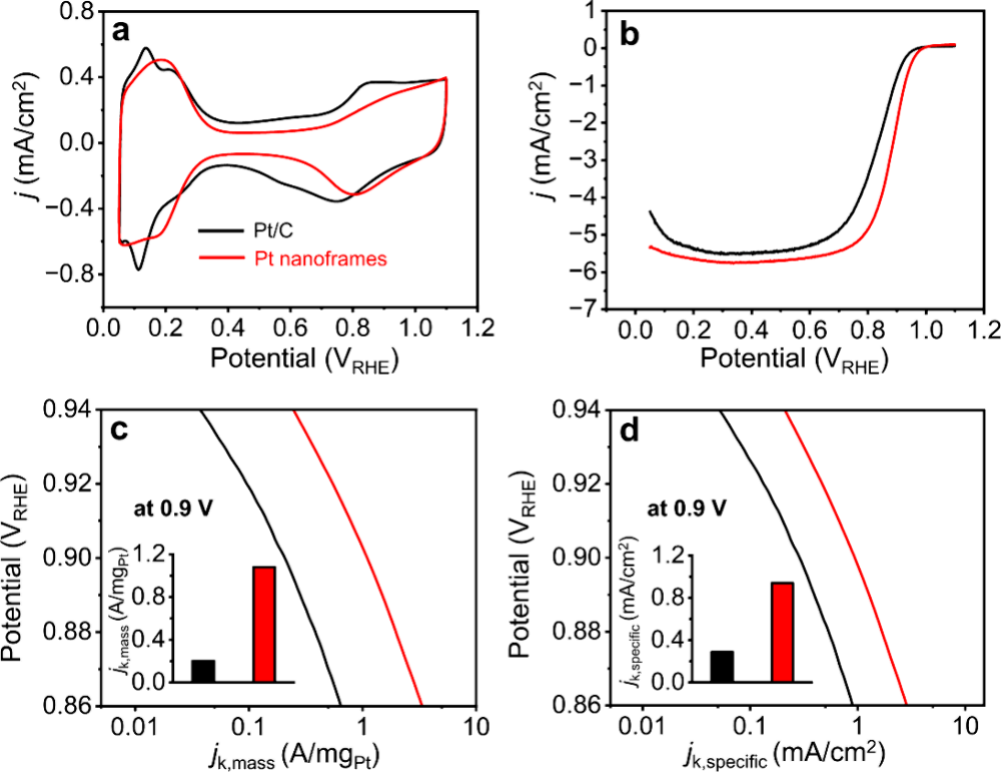
(a, b) CVs and positive-going ORR polarization curves recorded from the carbon-supported Pt icosahedral nanoframes and a commercial Pt/C catalyst, respectively. The currents were normalized to the geometric area (0.196 cm^2^) of the rotating disk electrode. (c, d) Plots of the specific and mass ORR activities given as kinetic current density (*j*_k_) normalized to the ECSA and Pt mass of the catalyst, respectively. The color scheme used in (a) applies to all panels.

The Pt icosahedral nanoframes also showed remarkable enhancement in catalytic activity toward the ORR, as confirmed by the positive-going ORR polarization curves in Figure 4b. The kinetic currents were then determined using the Koutecky–Levich equation. Specific activity (SA) and mass activity (MA) were derived by normalizing the kinetic current against the ECSA and the mass of Pt in the sample, respectively (Figure 4c, and d). Table S2 shows a summary of the MA and SA at 0.9 V_RHE_. Notably, the nanoframes had a high SA of 0.72 mA/cm^2^, which was more than two times as great as that of Pt/C. The high SA of the nanoframes could be attributed to the {111} facets and compressive strain on the surface. It is well documented that Pt{111} has an enhanced activity toward ORR relative to other facets typically found on the surface of Pt irregular nanoparticles found in Pt/C.^40^ On the other hand, compressive strain in Pt{111} could further enhance the SA by weakening the binding between the surface and OH. Because the Pt icosahedral nanoframes had both large specific ECSA and high SA, they gave a high MA of 0.83 A/mg_Pt_, which was four times as high as that (0.2 A/mg_Pt_) of the Pt/C.

We further evaluated the durability of the catalyst based on Pt icosahedral nanoframes through an accelerated durability test (ADT) by applying continuous cycling for up to 5000 cycles in an O_2_-saturated 0.1 M HClO_4_ solution. The peak areas corresponding to the desorption and adsorption of hydrogen in the CV curve exhibited a slight reduction, and the shape of the CV curve also changed after ADT (Figure 5a). Specifically, the shape of the CV curve became more similar to that of the pure Pt surface, suggesting that Pt atoms migrated to the surface due to the lower surface energy of Pt relative to Pd, thereby replacing the small fraction of Pd originally distributed on the surface.^28,41^ The Pd might also have been simply etched away, since it is more susceptible to oxidation and etching. There was almost no change to the current densities of the nanoframes, resulting in a stable SA of the nanoframes at 0.9 V_RHE_ after the ADT test (Figure 5b). Besides, TEM images of the nanoframes after ADT show that the icosahedral nanoframes maintained their {111} facets and there was no noticeable aggregation or detachment of particles (Figure 5c and d). Because the SA was highly stable and the ECSA only suffered a minor loss, the MA of the nanoframes also showed essentially no decay. The slightly increased SA can be attributed to the increased Pt composition on the surface. Because the ORR activity of Pt is higher than Pd, the SA of the nanoframes was slightly increased after the ADT test. Altogether, Pt icosahedral nanoframes exhibited enhanced activity and durability toward ORR, showing great promise as a future catalyst in fuel cell applications.

**Figure 5:**
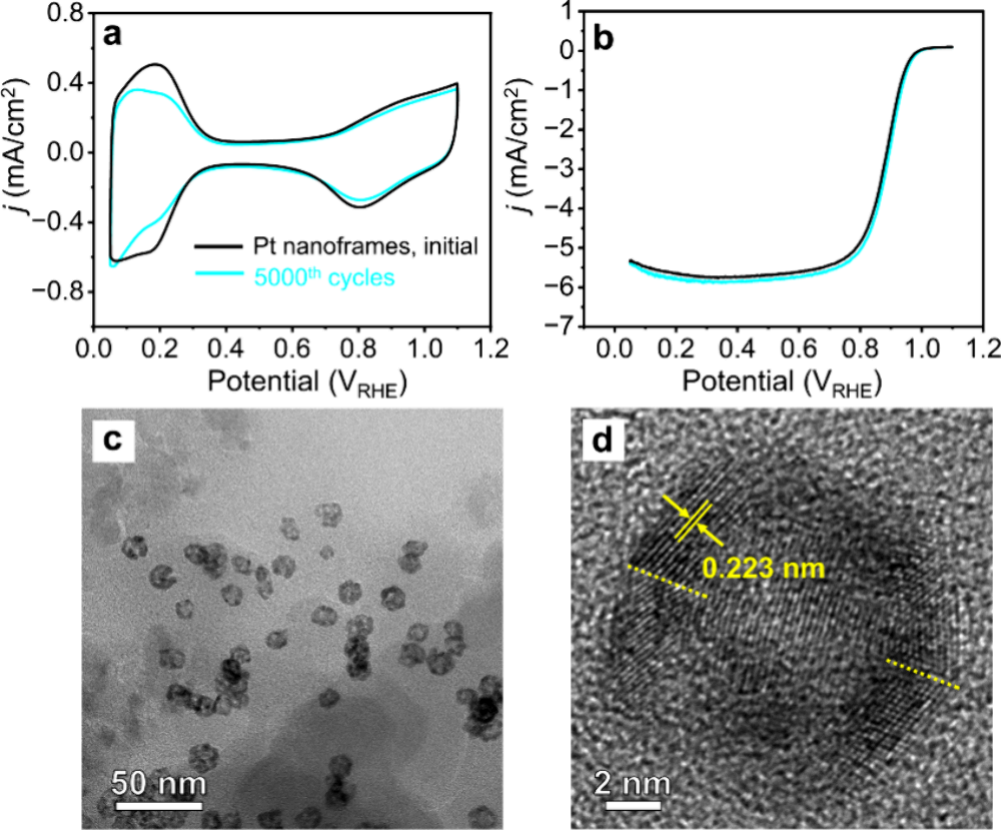
(a) CVs and (b) positive-going ORR polarization curves recorded from the carbon-supported Pt icosahedral at 0 and 5000 cycles. The currents were normalized to the geometric area (0.196 cm^2^) of the rotating disk electrode. (c) TEM and (d) HRTEM images of the carbon-supported Pt icosahedral nanoframes after 5000 cycles of ADT. The yellow dashed lines in (b) represent twin boundaries.

We demonstrated that the strain gradient on Pd icosahedra can be leveraged to achieve preferential oxidation and etching of Pd atoms from the facets and selective deposition of the resultant Pt atoms on the vertices and edges. Once the edges are covered by Pt atoms, the {111} facets become more favorable for oxidation and dissolution. Icosahedral nanoframes made of Pt will be obtained when essentially all of the Pd in the interior has been etched away. The Pt icosahedral nanoframes are found to replicate the twin boundaries on the vertices and edges of Pd icosahedra and feature compressively strained {111} facets. We also evaluate the catalytic activity and durability of the Pt icosahedral nanoframes toward ORR. The nanoframe-based catalyst shows a 4-fold enhancement in mass activity relative to the commercial Pt/C. Even after 5000 cycles of potential sweeping in 0.6–1.1 V_RHE_, the catalyst maintains its initial mass activity. The results collectively suggest that the Pt icosahedral nanoframes prepared by using the galvanic synthesis are promising as a substitute for the conventional Pt/C in fuel cell applications. The strain-controlled galvanic replacement also provides a synthetic route to the facile synthesis of nanocrystals with a compressive strain.

## Supporting Information Available

Strain distribution, size definition, SEM image, and STEM image of the Pd icosahedron; elemental compositions of the samples obtained at different reaction time points during galvanic replacement; SAs and MAs of Pt icosahedral nanoframes before and after ADT; TEM image of as-synthesized Pt icosahedral nanoframes on the carbon support (PDF)
