# Supplementary material for: Strain-Controlled Galvanic Synthesis of Platinum Icosahedral Nanoframes and Their Enhanced Catalytic Activity toward Oxygen Reduction
Source: Nano Lett. 2024 Oct 18;24(43):13513–9. doi: 10.1021/acs.nanolett.4c02764 (PMC11528434; doi:10.1021/acs.nanolett.4c02764)
Supplement: Supplementary file 1 — nl4c02764_si_001.pdf [file nl4c02764_si_001.pdf]

Supporting Information

Strain-Controlled Galvanic Synthesis of Platinum Icosahedral Nanoframes and Their Enhanced Catalytic Activity toward Oxygen Reduction

Siyu Zhou,[†] Minghao Xie,[‡] Yong Ding,[#] Zhiqi Wang,[‡] Quynh Nguyen,[‡] Kei Kwan Li,[‡] and Younan Xia[‡,§,†,*]

[†]School of Chemical and Biomolecular Engineering, Georgia Institute of Technology, Atlanta, Georgia 30332, United States

[‡]School of Chemistry and Biochemistry, Georgia Institute of Technology, Atlanta, Georgia 30332, United States

[#]School of Materials Science and Engineering, Georgia Institute of Technology, Atlanta, Georgia 30332, United States

[§]The Wallace H. Coulter Department of Biomedical Engineering, Georgia Institute of Technology and Emory University, Atlanta, Georgia 30332, United States

*Address correspondence to younan.xia@bme.gatech.edu

EXPERIMENTAL SECTION

Chemicals and Materials. Diethylene glycol (DEG, 99.0%, lot no. BCBJ9740), sodium tetrachloropalladate(II) (Na_2PdCl_4, 99.998%), potassium tetrabromopalladate(II) (K_2PdBr_4, 99.998%), potassium tetrachloroplatinate(II) (K_2PtCl_4, 98.0%), L-ascorbic acid (AA), ferric chloride ($FeCl_3$), potassium bromide (KBr), hydrochloric acid (HCl, 37%), acetic acid (99.7%), perchloric acid ($HClO_4$, 70%, PPT Grade, Veritas) and poly(vinyl pyrrolidone) (PVP, Mw ≈ 55,000) were all obtained from Sigma-Aldrich and used as received. Deionized (DI) water with a resistivity of 18.2 MΩ cm at room temperature was used throughout the experiments.

Synthesis of Pd icosahedra with an Average Size of 12 nm. We synthesized Pd icosahedra with an average size of 12 nm by following our previously published method.[1] In a standard protocol, 80 mg PVP was dissolved in 2 mL of DEG hosted in a 20-mL vial by heating the vial to 130 °C for 10 min under magnetic stirring. Afterwards, 1 mL of Na_2PdCl_4 solution (15.5 mg/mL, in DEG) was added in one shot. The reaction was allowed to proceed at 130 °C for another 3 h under magnetic stirring.

Seeded Growth of the 12-nm Pd Icosahedra. To enlarge the size of the icosahedra to 19 nm, 1 mL of K_2PdBr_4 solution (15.5 mg/mL, dissolved in DEG) was added into 1 mL of the reaction solution obtained from the last step. The reaction was allowed to proceed for 5 h. The solid products were crushed out using 6 mL of acetone, followed by two washes with ethanol. After each wash, the particles were collected through centrifugation. Finally, the particles were redispersed in 3 mL of water for further characterization and reaction.

Synthesis of Pt-Pd Icosahedral Nanoframes. In a typical synthesis, 0.1 mL of the suspension of 19-nm Pd icosahedra (obtained in the last step) was mixed with 4 mL of aqueous HCl (0.2 M) containing PVP (35 mg) and KBr (300 mg) in a 20-mL glass vial. The mixture was heated to 90 °C in air under magnetic stirring. Meanwhile, 2 mg of K_2PtCl_4 was dissolved in 2 mL of water. This K_2PtCl_4 solution was then injected into the preheated mixture containing PVP, KBr, and Pd icosahedra in one shot. The reaction was allowed to proceed at 90 °C for 15 h in air under magnetic stirring. Finally, the solid products were collected by centrifugation. After washing three times with water to remove excess PVP, the particles were dispersed in 1 mL of water.

Synthesis of Pt Icosahedral Nanoframes. The Pd component was removed from the Pt–Pd icosahedral nanoframes *via* chemical etching. In a typical process, 300 mg of KBr, 50 mg of PVP, 10 mg of $FeCl_3$, 0.06 mL of HCl, and 5.94 mL of water were mixed in a 20-mL glass vial. This mixture was held at 90 °C in an oil bath under magnetic stirring and 1 mL of the

aqueous suspension of Pt-Pd icosahedral nanoframes (the concentration of Pd: 0.86 mg mL^{-1}) was introduced. After etching for 2 h, the solid products were collected by centrifugation, followed by washing three times with ethanol and water.

Morphological, Structural, and Elemental Characterizations. Transmission electron microscopy (TEM) analysis was performed on an HT7700 microscope (Hitachi) operated at 120 kV. The elemental compositions of the samples were determined using an inductively-coupled plasma mass spectrometer (ICP–MS, NexION 300 Q, PerkinElmer). High-resolution TEM (HRTEM) images, scanning transmission electron microscopy (STEM) images, and energy-dispersive X-ray (EDX) spectroscopy data were acquired on a transmission electron microscope (FEI Technai F30) equipped with a EDX detector. The sample for the electron microscopy was prepared by placing a drop of particle suspension on the Cu grid, followed by drying in the air.

Preparation of the Working Electrodes. Firstly, the Pt icosahedral nanoframes were loaded on a carbon support (Vulcan XC-72) with a Pt loading content of 9 wt %. Then, the carbon-supported nanoframes (the catalyst) were dispersed in 6 mL of acetic acid and heated at 60 ºC for 3 h to help clean the surface. The catalyst was retrieved by centrifugation and washed three times with ethanol, followed by drying in an oven at 80 ºC for 2 h. To produce the ink, 3 mg of the catalyst was re-dispersed in a mixture of 1 mL of water, 1 mL of isopropanol, and 15 μL of 5% Nafion under ultrasonication for 20 min. The Pt concentration was determined to be 0.12 mg/mL by ICP-MS. To prepare the working potential, 8 μL of catalyst ink was drop cast on a pre-cleaned glassy carbon rotating disk electrode (RDE, Pine Research Instrumentation). The Pt/C commercial catalyst (3.2-nm Pt particles supported on the Vulcan XC72 carbon, Premetek Co., with a Pt loading of 20 wt %) served as a benchmark for electrocatalytic activity. The ink was prepared by mixing 2 mg of the Pt/C catalyst, 1 mL of water, 1 mL of isopropanol and 10 μL of 5% Nafion under ultrasonication for 20 min. The Pt concentration in the ink was determined to be 0.17 mg/mL by ICP-MS. To prepare the working electrode, 6 μL of the ink was deposited on pre-cleaned RDE, followed by drying under ambient conditions and at room temperature.

Electrochemical Measurements. The electrochemical measurement was performed in a three-electrode system controlled by a potentiostat (CHI 600E, CH Instruments) at room temperature. Platinum mesh and reversible hydrogen electrode (RHE) served as the counter and reference electrodes, respectively. To obtain the electrochemical active surface area (ECSAs), we first recorded the cyclic voltammogram (CV) curves in the Ar-saturated

electrolyte (0.1 M $HClO_4$) by cycling between 0.05–1.1 V_{RHE} at a sweeping rate of 0.05 V/s. Then, we calculated the average charges (Q_H) associated with the adsorption of hydrogen from 0.05−0.4 V_{RHE} after double-layer correction and determined the ECSAs by assuming 210 $\mu C/cm^2$ (for commercial Pt/C) or 240 $\mu C/cm^2$ (for nanoframes) for the adsorption of a monolayer of hydrogen from Pt surfaces. We carried out the oxygen reduction tests at room temperature in the potential range of 0.05–1.1 V_{RHE} in a O_2-saturated $HClO_4$ solution (0.1 M) at a scan rate of 0.01 V/s and a rotating speed of 1600 rpm. The data were corrected by 95% ohmic *i*R drop compensation. The accelerated durability tests were carried out in the range of 0.6–1.1 V_{RHE} for 5000 cycles at a rate of 100 mV/s in the O_2-saturated $HClO_4$ solution (0.1 M). The kinetic current at 0.9 V_{RHE} was calculated based on the Koutecky–Levich equation. Mass and specific activities of the samples were obtained by normalizing the kinetic current at 0.9 V_{RHE} to the Pt loadings and ECSAs, respectively.

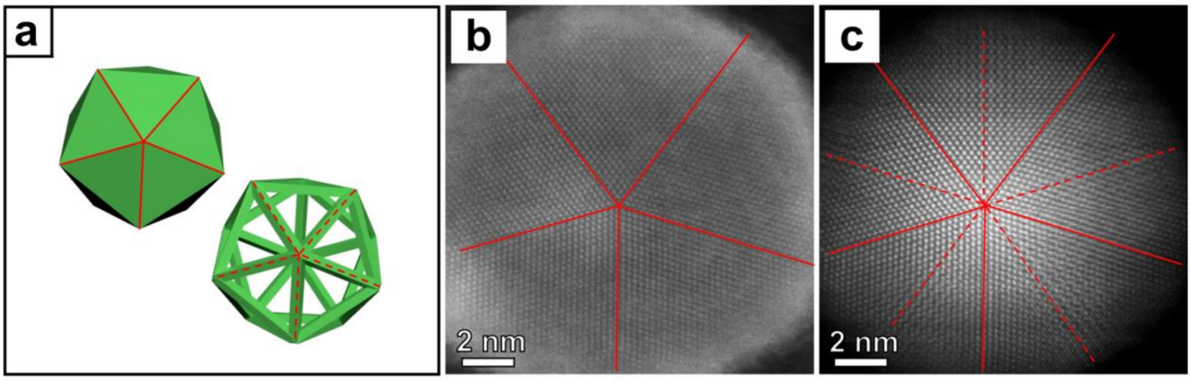

Figure S1. (a) Model, (b) SEM image, and (c) STEM image of an icosahedron oriented with its five-fold axis along the electron beam. Note that the two models in (a) correspond to the same icosahedron except that the frame-like model only shows the vertices and edges to elucidate the positions of twin boundaries on the side facing away from us. In the corresponding SEM image (b), a five-fold symmetry is resolved on the side facing us. In the STEM image (c), except for the five-fold symmetry shown in (b), another one is resolved on the side facing away from us. The red solid lines represent the twin boundaries on the side facing us, while the red dashed lines represent the twin boundaries on the side facing away from us.

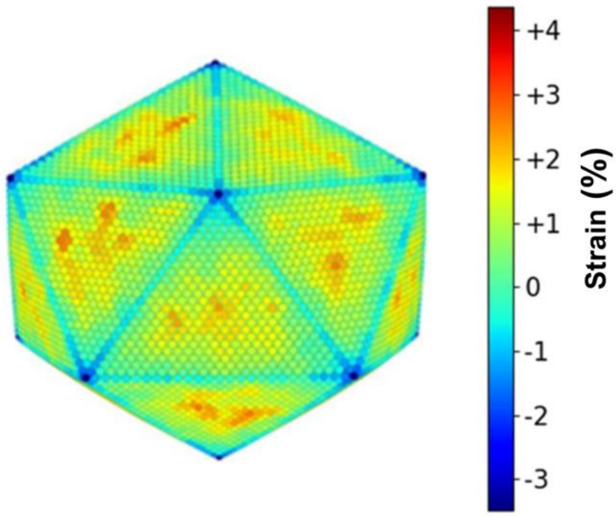

Figure S2. Calculated strain distribution on the surface of a Pd icosahedral nanocrystal. Modified with permission from ref 1. Copyright 2023 Wiley-VCH.

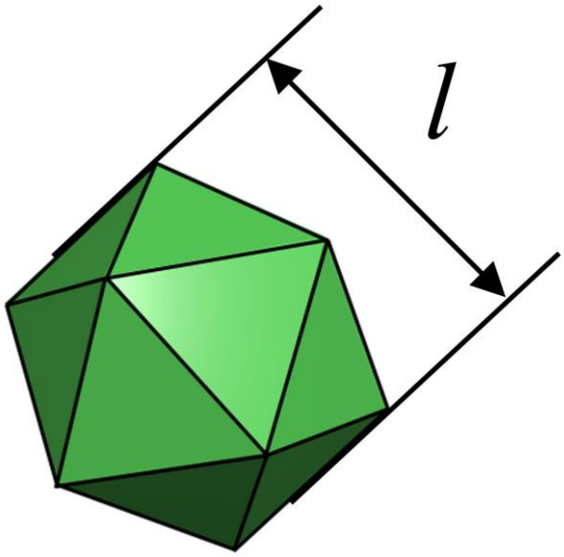

Figure S3. Schematic showing how the size of an icosahedral nanocrystal is defined.

Table S1. The evolution of Pt/Pd ratio with reaction time during the galvanic replacement between Pd icosahedra and K_2PtCl_4 in the presence of KBr.

**Reaction time**	0 h	1 h	15 h
**Pt/Pd ratio**	0	0.65	1.04

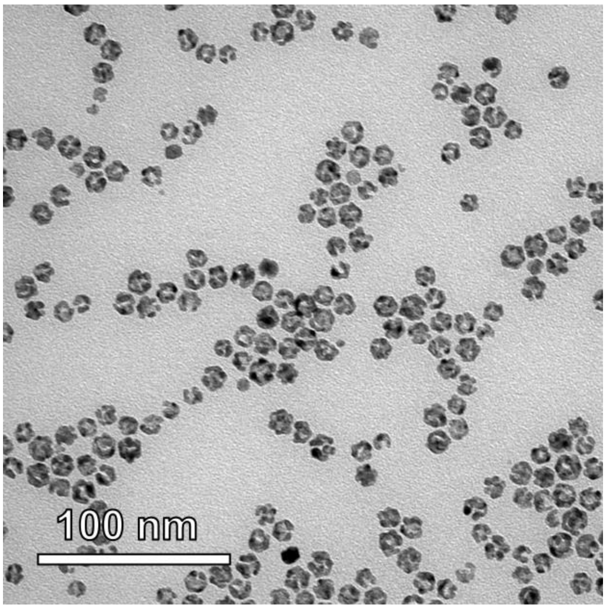

Figure S4. TEM image of Pt-Pd nanoframes obtained using the standard protocol except that the Pd icosahedra had a size of 10 nm.

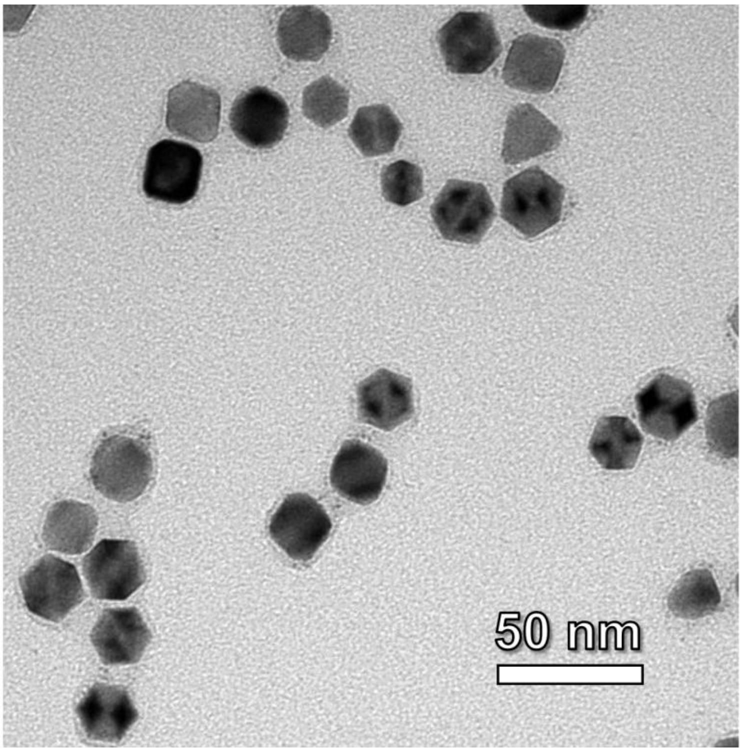

Figure S5. TEM image of the sample obtained using the standard protocol without KBr.

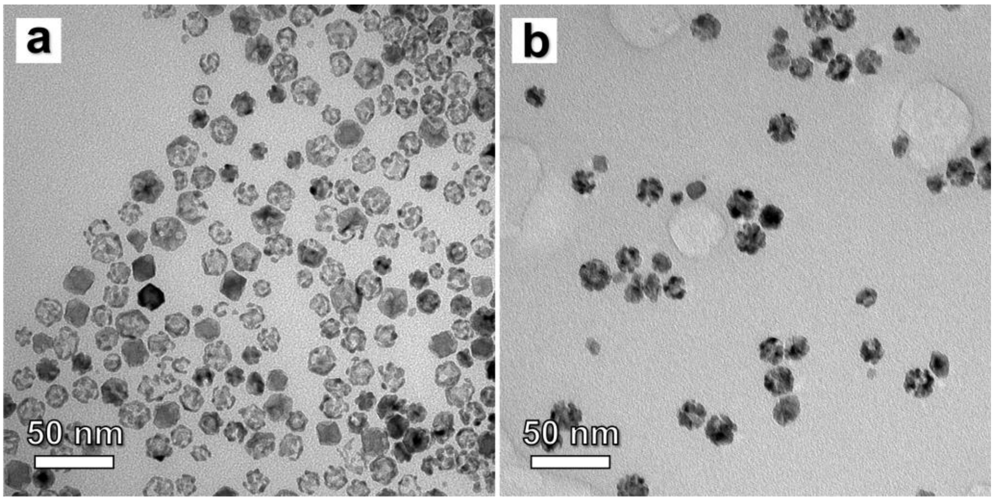

Figure S6. TEM images of Pt-Pd nanoframes obtained using the standard protocol except for modifications to the concentration of Pd icosahedra: (a) halving the concentration, and (b) doubling the concentration, respectively.

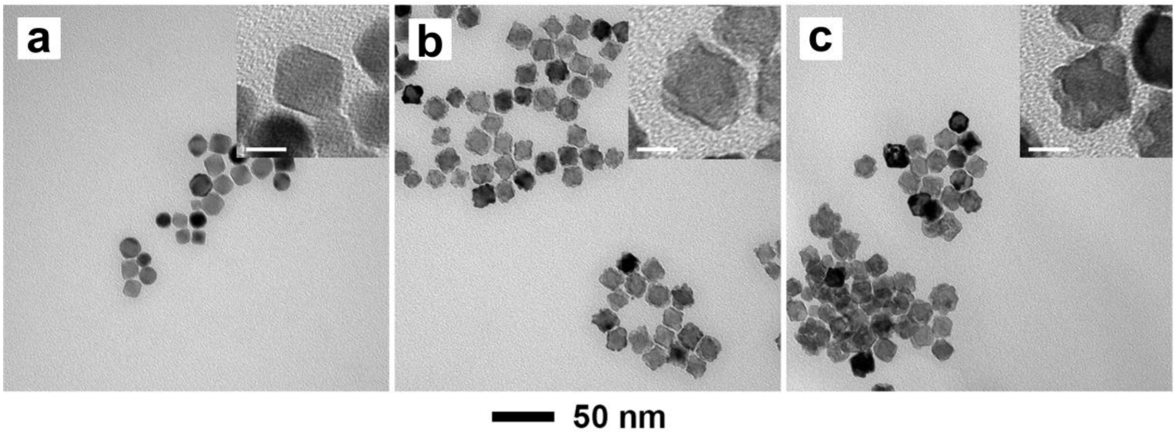

Figure S7. TEM images of the samples obtained using the standard protocol except for the replacement of Pd icosahedra with the same amount of Pd octahedra, and the reaction was allowed to proceed for: (a) 0, (b) 1, and (c) 15 h.

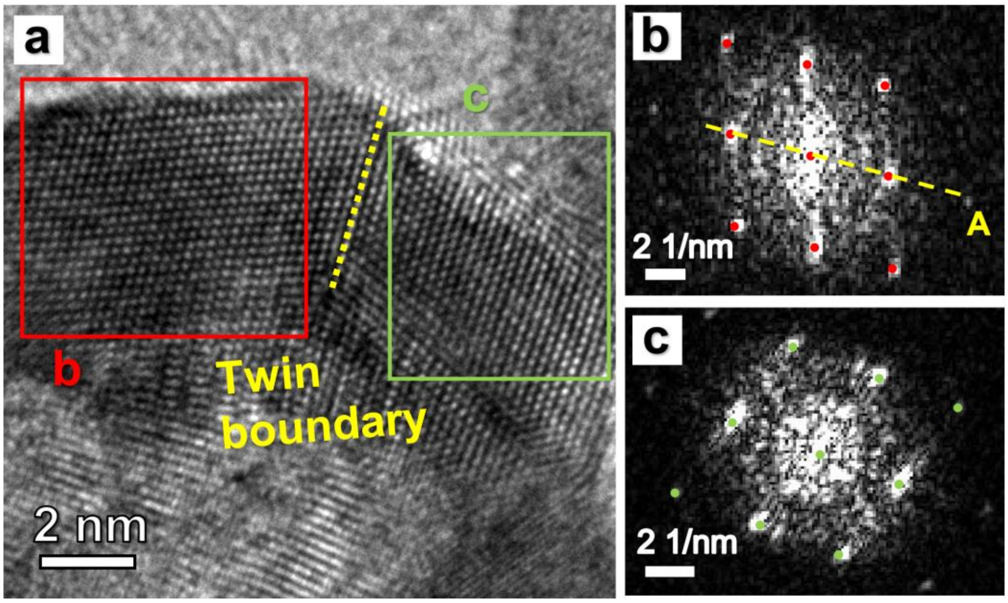

Figure S8. (a) HRTEM image of a Pt-Pd nanoframe taken out from a sample prepared using the standard protocol. (b,c) FFT patterns of the Pt-Pd nanoframe corresponding to different regions shown in (a).

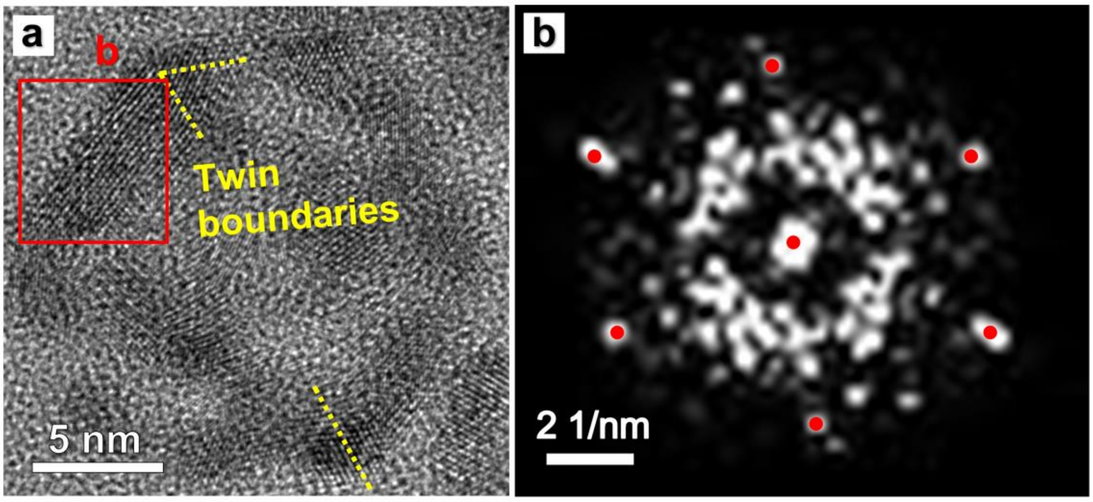

Figure S9. (a) HRTEM image of a Pt nanoframe taken from the sample prepared using the standard protocol. (b) FFT pattern of the Pt nanoframe corresponding to boxed region in (a).

Table S2. Electrochemically active surface area (ECSA), mass activity (MA), and specific activity (SA) of the Pt icosahedral nanoframes before and after 5000 cycles of the accelerated durability test and commercial Pt/C catalysts.

**Samples**	**Cycles**	**ECSA**	**MA**	**SA**
		$m^2\ g^{-1}{}_{Pt}$	$A\ mg^{-1}{}_{Pt}$	$mA\ cm^{-2}$
Pt icosahedral nanoframes	Initial	115.2	0.83	0.72
	5000	103.1	0.81	0.79
Commercial Pt/C	Initial	69.4	0.20	0.29

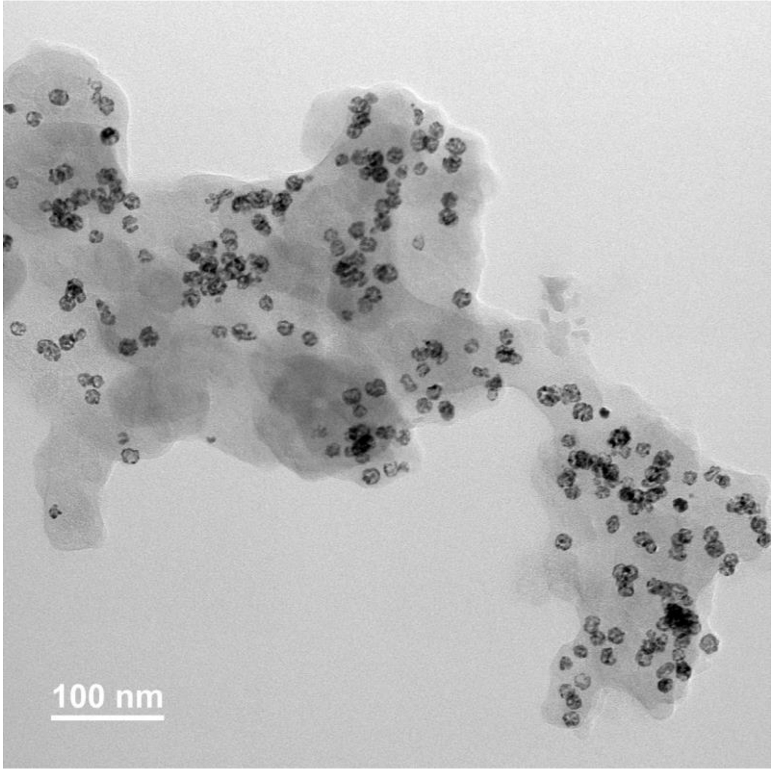

Figure S10. TEM image of the as-synthesized Pt icosahedral nanoframes supported on carbon.

REFERENCES

(1) Zhou, S.; Figueras-Valls, M.; Shi, Y.; Ding, Y.; Mavrikakis, M.; Xia, Y. Fast and Non-equilibrium Uptake of Hydrogen by Pd Icosahedral Nanocrystals. *Angew. Chem. Int. Ed.* **2023**, *62*, e202306906.
